# Interkingdom interaction: the soil isopod Porcellio scaber stimulates the methane-driven bacterial and fungal interaction

**DOI:** 10.1038/s43705-023-00271-3

**Published:** 2023-06-24

**Authors:** Tanja Heffner, Semi A. Brami, Lucas W. Mendes, Thomas Kaupper, Emilia S. Hannula, Anja Poehlein, Marcus A. Horn, Adrian Ho

**Affiliations:** 1 Leibniz Universität Hannover, Institute for Microbiology, Herrenhäuser Str. 2, 30419 Hannover, Germany; 2 University of São Paulo CENA-USP, Center for Nuclear Energy in Agriculture, Avenida Centenario, 303, 13416-000 Piracicaba (SP), Brazil; 3 Leiden University, Department of Environmental Biology, Institute of Environmental Sciences, Einsteinweg 2, 2333CC Leiden, the Netherlands; 4 Georg-August University Göttingen, Department of Genomic and Applied Microbiology and Göttingen Genomics Laboratory, Institute of Microbiology and Genetics, Grisebachstr. 8, D-37077 Göttingen, Germany

**Keywords:** Microbial ecology, Microbial ecology, Biogeochemistry

## Abstract

*Porcellio scaber* (woodlice) are (sub-)surface-dwelling isopods, widely recognized as “soil bioengineers”, modifying the edaphic properties of their habitat, and affecting carbon and nitrogen mineralization that leads to greenhouse gas emissions. Yet, the impact of soil isopods on methane-cycling processes remains unknown. Using *P. scaber* as a model macroinvertebrate in a microcosm study, we determined how the isopod influences methane uptake and the associated interaction network in an agricultural soil. Stable isotope probing (SIP) with ^13^C-methane was combined to a co-occurrence network analysis to directly link activity to the methane-oxidizing community (bacteria and fungus) involved in the trophic interaction. Compared to microcosms without the isopod, *P. scaber* significantly induced methane uptake, associated to a more complex bacteria-bacteria and bacteria-fungi interaction, and modified the soil nutritional status. Interestingly, ^13^C was transferred via the methanotrophs into the fungi, concomitant to significantly higher fungal abundance in the *P. scaber*-impacted soil, indicating that the fungal community utilized methane-derived substrates in the food web along with bacteria. Taken together, results showed the relevance of *P. scaber* in modulating methanotrophic activity with implications for bacteria-fungus interaction.

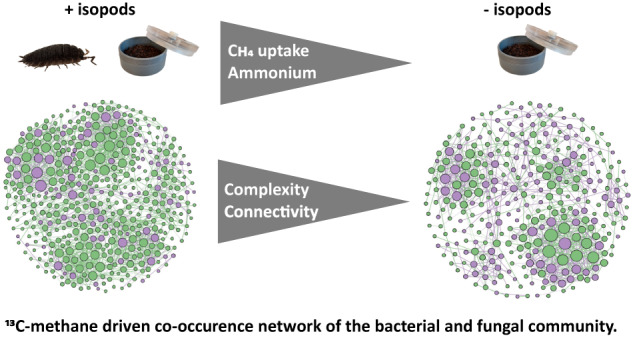

## Introduction

Macroinvertebrates are integral members of the soil biota. The activity of macroinvertebrates not only re-distributes resources and modifies the soil physico-chemical properties influencing the soil structure and spatial heterogeneity, but also affects the soil microorganisms [1–4]. By engineering their habitat, macroinvertebrates may impose an indirect long-term effect in re-shaping the microbial community composition via bioturbation [5], with consequences for microbially-mediated soil processes [6, 7]. Also, macroinvertebrates may passively disperse microbial propagules, transporting microorganisms which have restricted mobility across large areas through their gut passage (fecal deposition; [8, 9]) and/or via their body surface. As part of the soil food web, macroinvertebrates can profoundly impact diverse aspects of microbial life in soils.

Bioturbation-induced impact on the soil fungal and bacterial communities have been studied independently, and shown to exert (in)direct differential effects on both these microbial groups [10–13]. Depending on the macroinvertebrate, selective grazing by millipedes, and nematodes may reduce fungal biomass and modifies the composition of fungal communities [10, 11], whereas the presence of other macroinvertebrates (e.g., earthworms) increases the bacterial biomass [12]. In particular, previous work focused primarily on the response of generalized fungal- or bacterial-mediated soil processes (e.g., respiration, C- and N-mineralization, enzymatic activity; [4, 6, 7, 14]) to the presence of macroinvertebrates, with the majority of earlier studies emphasized on earthworms below ground [14–17]. Yet, many macroinvertebrates are (sub-)surface dwellers, among which, *P. scaber* (common woodlouse, family *Porcellionidae*) is a cosmopolitan soil isopod, albeit native to Europe [18]. *P. scaber*, together with other soil isopods, significantly contribute to the decomposition of leaf litter, consuming up to 10% of the annual litterfall in grasslands [7, 19]. Specifically, *P. scaber* activity (e.g., feeding and foraging habits, fecal deposition) may also induce compositional shifts in the soil microbial community, having an indirect effect on microbially-mediated organic matter decomposition [4]. Although recent work began to shed light on microbe-macroinvertebrate interaction in terrestrial ecosystems [20], virtually nothing is known on how (sub-)surface-dwelling macroinvertebrates exert an impact on other non-CO_2_ primary greenhouse gases, particularly methane.

In this respect, methane consumption in well-aerated soils is predominantly catalyzed by the aerobic methane-oxidizers (methanotrophs), a microbial guild with low diversity, comprising of members belonging to Verrucomicrobia, Actinobacteria, and Proteobacteria (gamma- and alpha-proteobacteria; [21–23]). While the verrucomicrobial methanotrophs may show habitat preference, inhabiting acidic and thermophilic geothermal environments and volcanic soils [24, 25], the proteobacterial methanotrophs are ubiquitous, and are relevant methane sinks in diverse terrestrial ecosystems [26–28]. The gamma- and alpha-proteobacterial methanotrophs can be further distinguished based on their carbon assimilation pathway, biochemistry, and ecophysiology, reflecting on their life strategies and response to perturbations [29–32]. Because the methanotrophs inhabit a specific niche where their main substrates, methane and oxygen are available (e.g., surface-top soil), these bacteria, as well as other (micro)organisms relying on the methane-derived carbon, are potentially influenced by the activity of soil isopods like *P. scaber*.

Here, we elucidated the response of the methane-driven fungal and bacterial communities and their interaction to *P. scaber* bioturbation. Because of the high variability in microbial responses to different macroinvertebrates, their distinct behavior patterns, and mode of interaction with microorganisms in naturally-occurring communities (e.g., grazing, burrowing activity, fecal deposition; [33–36]), *P. scaber* was used as the model organism in a proof-of-principle study to minimize these confounding effects. Coupling DNA-based stable isotope probing (SIP) using ^13^C-methane to a co-occurrence network analysis (incorporating both the metabolically active fungal and bacterial communities), we directly linked the response of the methanotrophic activity (methane uptake) to the structure of the fungal and bacterial interaction networks (i.e., methane-based food web) to determine how *P. scaber* influences members of the active soil microbiome. Here, we defined the methane-driven interaction network as the methanotroph “interactome”, a subpopulation of the community comprising of the methanotrophs and non-methanotrophs that could be tracked via the flow of the methane-derived ^13^C [37, 38]. Accordingly, we hypothesized that bioturbation promotes methanotrophic activity and growth as a consequence of comminution/physical re-organization of the soil, which increases aeration, in turn stimulating aerobic processes, including methane oxidation. Moreover, *P. scaber* may aid in the dispersal and transport of microorganisms, increasing the habitat range of soil microorganisms, and thus shaping the microbial community composition and soil processes in the newly colonized environment.

## Methods

### Soil and soil sampling, and establishing a P. scaber colony

The soil (upper 10 cm) was collected in August 2016 from the fringe of an agricultural field in Vredepeel (51^o^32´32´´N, 05^o^50´54´´E) belonging to the Wageningen University and Research (the Netherlands). *P. scaber* was observed in the sampling site, which was strewn with stones/rocks and harvested crop residues. The apparent K_M_ and V_max_ of this soil are indicative of “low-affinity” methane oxidation, albeit oxidation at circum-atmospheric methane concentrations (≤40–50 ppm_v_) has also been documented [39, 40]. After sampling, the soil (sandy loam) was air-dried at ambient temperature, sieved (2 mm) to remove debris (stones and wood) and roots, and stored in an enclosed plastic container at room temperature till microcosm setup. An aliquot of the air-dried and sieved soil was sterilized by γ-irradiation (25 kGy; ^60^Co) [41]. The soil pH was 5.4, and contained a total C and N of 22.2 µg C mg dw^−1^ and 1.3 µg N mg dw^−1^, respectively. Other selected soil physico-chemical parameters have been reported elsewhere [42, 43]. The soil was fallow during sampling, but was cropped with potatoes prior to sampling.

*P. scaber* was collected from under potted plants and rocks around the Institute for Microbiology, Leibniz University Hannover, Germany (52^o^23´36´´N; 9^o^42´17´´E). The isopods were visually inspected to ensure that only the same species with a body length of >0.5–1.0 cm were collected. In parallel, leaf litter, mainly comprising of *Quercus rubra* (~95%) and *Calystegia sylvatica* (~5%), was collected from the sampling site to serve as feed for the isopod [44]. The leaf litter was dried and coarsely blended to achieve litter fragments with comparable sizes before being autoclaved. Eighty isopods were collected into a glass petri dish (20 cm diameter; 4.5 cm height) with a lid (non-gas tight) containing the sandy loam soil moistened with autoclaved deionized water (50% gravimetric water content), wood chips/tree bark, stones, and leaf litter to establish a *P. scaber* colony (i.e., a reservoir of *P. scaber* to replace dead/moribund individuals in the subsequent microcosm study). The leaf litter was replenished weekly, while the enclosure was sprayed with water every 3–4 days. The isopods were acclimatized under the experimental conditions in the reservoir for at least one week prior to microcosm setup.

### Microcosm setup

To determine the effects of *P. scaber* bioturbation on the methanotrophic activity and soil microbial community, microcosms in the presence and absence of the isopods were compared. Each microcosm consisted of 0.5 g autoclaved leaf litter and 20 g dry-weight soil saturated with 4 mL autoclaved deionized water in a Petri dish. Two uniformly sized *P. scaber* were added into the microcosm ( + isopod; *n* = 6) approximating the field density per area [45] to be compared to microcosms without isopods (- isopod; *n* = 6). Prior to addition, the isopod was lightly rinsed with deionized water to dislodge loosely attached soil particles. In the microcosms containing *P. scaber*, dead isopods (see Tables S1, S2) were replaced with individuals of a similar size from the reservoir throughout the incubation. A microcosm without *P. scaber* and leaf litter, containing only soil, served as a reference (*n* = 4). Additional microcosms were constructed with the exception that γ-irradiated soil was used to determine whether the isopods may act as microbial propagules; these microcosms (*n* = 6) were destructively sampled over time after 14 (*n* = 3) and 49 days (*n* = 3) incubation.

After setup, the microcosms were placed in gas-tight flux chambers [46]. In the microcosms with (+isopod) and without (- isopod) *P. scaber*, the headspace gas was adjusted to ~ 4%_v/v_ methane (^13^C-CH_4,_ *n* = 4 and ^12^C-CH_4_, n = 2 each for the + and - isopods) in air, while unlabeled methane (^12^C-CH_4_) was used for the other incubations. The headspace methane was replenished when dead isopods were replaced or methane/oxygen was depleted. Hence, cumulative methane and carbon dioxide fluxes were determined by consecutively adding the total methane consumed or carbon dioxide produced whenever the headspace gas was replenished. The incubation was performed in the dark at 27 °C until >400 µmole methane was consumed to ensure sufficient ^13^C labeling [38]. After the incubation, the isopod was removed and the soil was homogenized. An aliquot of the soil was stored in the −20 °C freezer till DNA extraction, and the remaining sample was used to determine the soil physico-chemical properties.

Additionally, 0.5 g autoclaved leaf litter was incubated in a 120 ml bottle capped with a butyl rubber stopper under the same conditions as in the microcosm setup (at 27 °C in the dark) to determine whether the leaf litter emits volatile organic compounds (VOCs). VOCs in the headspace of the incubation were compared to concentrations at room atmosphere. Emitted volatile compounds were below the detection limit.

### Analytical methods

The headspace methane and carbon dioxide were measured using a gas chromatograph (7890B GC System, Agilent Technologies, Santa Clara, USA) coupled to a pulsed discharge helium ionization detector (PD-HID), with helium as the carrier gas [47]. The rate of methane uptake was determined from the slope (linear regression) of the cumulative methane uptake curve. The soluble ammonium, nitrate, and nitrite concentrations were determined colorimetrically in deionized water (1:5 _w/v_) following centrifugation and filtration (0.2 µm) of the soil suspension [48, 49] using an Infinite M plex plate reader (TECAN, Meannedorf, Switzerland). Nitrate and nitrite concentrations were below the detection limit (<3–4 µM). VOCs were measured by gas chromatography (Agilent 7890 GC System) coupled to a flame ionization detector and mass spectrometry (Agilent 5977B mass spectrometer; Santa Clara, USA). Samples were injected at 60 °C injector starting temperature (splitless injection; Gerstel MPS robotic sample manager and Gerstel CIS cooled injection system; Mühlheim an der Ruhr, Germany) with a 12 °C temperature gradient till 360 °C, and separated on a 30 m × 0.25 mm column with a 0.25 µm film using the following temperature program: 30 °C for 1 min, increasing to 200 °C at 10 °C per min, final hold time of 2 min. Helium 6.0 with constant gas flow of 1.25 ml per min and initial pressure of 14.806 psi was used as carrier gas and volatiles detected at 70 eV in electron ionization mode. Electrical conductivity (EC) and pH were respectively measured using an EC electrode (Groline H198331, HANNA Instruments, RI, USA) and pH meter (METTLER TOLEDO, Giessen, Germany). EC was low (<0.03 dS m^−1^), and pH was within a narrow range (6.4–6.7) during the incubation.

### DNA extraction and isopycnic ultracentrifugation for DNA-based stable isotope probing (SIP)

DNA extraction was performed in triplicate for each sample and pooled after elution to obtain sufficient nucleic acid, as detailed before [50], with minor modifications [51]. The DNA was extracted from the starting material, and after all incubations for SIP and quantification of specific gene abundances (qPCR).

The DNA-based SIP was performed as described before [52] for incubations with (+isopod) and without isopods (- isopod). Briefly, isopycnic ultracentrifugation was performed for 67 h at 144,000 × *g* using an Optima L-80XP ultracentrifuge (Beckman Coulter Inc., USA). The ultracentrifugation run was performed with DNA extracted from incubations containing ^13^C- and ^12^C-CH_4_ in parallel to distinguish the “heavy” fraction (^13^C-enriched DNA) from the “light” fraction (unlabeled DNA) (Fig. S1). Following ultracentrifugation, fractionation was immediately performed using a peristaltic pump (Duelabo, Dusseldorf, Germany) at a rate of 2.8 rpm min^−1^. In total, 9–10 fractions were obtained per sample after discarding the last fraction. The density gradient of each fraction was determined using an AR200 digital refractometer (Reichert Technologies, Munich, Germany). Thereafter, the DNA was precipitated over night as described elsewhere [52], before undergoing two washing steps with ethanol, and suspended in 30 µL ultrapure PCR water (INVITROGEN, Waltham, USA). A qPCR assay (see below) targeting the *pmoA* gene was applied to the DNA from each fraction to identify the “heavy” and “light” fractions after comparing the DNA derived from the ^13^C- and ^12^C-CH_4_ incubations (Fig. S1). Identification of the “heavy” and “light” fractions were as defined before [52]; the internal transcribed spacer (ITS) and 16 S rRNA gene from these fractions were amplified for Illumina MiSeq sequencing to construct the co-occurrence networks.

### ITS-, 16 S rRNA-, and pmoA gene-targeted qPCR assays

The qPCR assays targeting the ITS, 16 S rRNA and *pmoA* (encoding the particulate methane monooxygenase, pMMO) genes were performed to enumerate the fungal, bacterial, and methanotroph abundances, respectively. The qPCR was performed using a BIORAD CFX Connect RT System (Biorad, Hercules, USA). The reagents, reagent concentrations, and PCR thermal profile for the qPCR assays targeting the 16 S rRNA (primer pair, 341 F/805 R) and *pmoA* (primer pair, A189f/mb661r) genes were as performed before [28], with the exception that the DNA template for the 16 S rRNA- and *pmoA*-based assays were diluted 50- and 20-folds, respectively. Note that the DNA template from the density gradient fractions for the *pmoA*-based qPCR was not diluted (Fig. S1). Each qPCR reaction targeting the ITS (total volume, 20 µL) consisted of 10 µL 2X SensiMix SYBR & Fluorescein Kit (Meridian Bioscience Inc., OH, USA), 1 µL of ITS3/ITS4 primer each (10 µM), 1 µL 0.04% BSA, 5 µL PCR-grade water, and 2 µL template DNA (diluted 20-fold). In a preliminary qPCR run, the dilutions used of the DNA template yielded the maximum gene copy numbers. The calibration curve for the qPCR assays (10^2^–10^8^ copy number of target genes) were derived from gene libraries, as described elsewhere [53]. The specificity of the amplicon was confirmed from the melt curve and by 1% agarose gel electrophoresis showing a single band of the correct size.

### Amplification of the ITS and 16 S rRNA gene for Illumina MiSeq sequencing

The ITS and 16 S rRNA gene were amplified using tagged primer pairs ITS3/ITS4 and 341 F/805 R, respectively. Each PCR reaction (total volume, 40 µl) comprised of 20 µl 2 X KAPA HIFI (Roche, Basel, Switzerland), 2 μl forward and reverse tagged-primers each (10 μM), 2 μl BSA (1%), 2 μl template DNA and 12 μl PCR-grade water. The PCR thermal profile targeting the ITS consisted of an initial denaturation step at 95 °C for 3 min, followed by 35 cycles of denaturation at 98 °C for 15 s, annealing at 55 °C for 15 s, and elongation at 72 °C for 15 s. The final elongation step was at 72 °C for 1 min. The PCR thermal profile targeting the 16 S rRNA was the same as in the PCR targeting the ITS, with the exception that 30 PCR cycles were performed, and the annealing temperature was at 53 °C. After confirming the specificity of the ITS and 16 S rRNA gene amplicons by 1% agarose gel electrophoresis, the PCR products were purified using the GeneRead Size Selection Kit (Qiagen, Hilden, Germany). The purified amplicons were used as template for a subsequent PCR to attach the adapters using the Nextera XT Index Kit (Illumina, San Diego, USA). The second PCR reaction for both genes are the same, and consisted of 12.5 µl 2X KAPA HiFi HotStart Ready Mix (Roche, Mannheim, Germany), 2.5 µl of each tagged primers (10 μM), 2.5 µl PCR grade water, and 5 µl template from the first PCR. Next, the amplicons were purified using the MagSi-NGS^PREP^ Plus Magnetic beads (Steinbrenner Laborsysteme GmbH, Wiesenbach, Germany), as described by the manufacturer. Normalization of the amplicons (equimolar amounts, 133 ng per sample) was performed using the Janus Automated Workstation (Perkin Elmer, Waltham, Massachusetts, USA) for library preparation and sequencing using Illumina MiSeq version 3 chemistry (paired-end, 600 cycles).

### ITS and 16 S rRNA gene amplicon analyses

The paired-end reads for both the ITS and 16 S rRNA gene sequences were merged using PEAR [54] and subsequently processed using QIIME 2 version 2021.21. De-multiplex and quality control were performed with DADA2 [55] by applying the consensus method to remove chimeric and low-quality sequences. For the 16 S rRNA gene sequence dataset, approximately 1 273 000 sequences were obtained after filtering, with an average of ~ 31,050 sequences per sample. After removing singletons and doubletons, the samples were normalized to 17,540 sequences, following the sample with the lowest number of sequences. Taxonomic identification was performed at 97% sequence similarity based on the Silva database v. 132 [56], and was given here to the finest resolution (genus/species level), whenever available. An average of 470 and 317 bacterial OTUs were derived from the ^13^C-enriched fraction, representing ~37% and ~28% of the total OTUs in the microcosms with and without isopods, respectively. For the ITS dataset, approximately 2 038 500 sequences were obtained after filtering (on average, ~ 49,700 sequences per sample), and were normalized to the lowest number of sequences per sample (34,920 sequences) after removing singletons and doubletons. Taxonomic identification was performed at 97% similarity based on the UNITE database [57]. An average of 60 and 61 fungal OTUs were derived from the ^13^C-enriched fraction, representing ~45% and ~38% of the total OTUs in the microcosms with and without isopods, respectively. A principal component analysis (PCA) was performed in Canoco 5 (Biometrics, Wageningen, the Netherlands) to visualize differences in the ^13^C- and ^12^C-derived fungal and bacterial community composition, whereas a redundancy analysis (RDA) was performed to determine the response of the active (i.e., ^13^C-derived) fungal and bacterial communities to the presence of *P. scaber* and to identify the abiotic parameters influencing the composition of the community. The PCA and RDA were based on the relative abundances of the ITS and 16 S rRNA gene. The data matrix was first analyzed using the detrended correspondence analysis (DCA) to determine whether the best-fit mathematical model was a PCA (for linearly distributed data) or RDA. Moreover, a permutational multivariate analysis of variance (PERMANOVA; [58]) was performed using PAST 4 software [59] to determine whether the composition of the fungal and bacterial communities in the treatments was significantly different. The ITS and 16 S rRNA gene sequences were deposited at the National Center for Biotechnology Information (NCBI), SRA database under the BioProject accession number PRJNA944204.

### ITS- and 16 S rRNA gene-based co-occurrence network analysis

A co-occurrence network analysis was performed, combining both the fungal and bacterial taxa (i.e., OTU-level, ITS and 16 S rRNA gene) derived from the ^13^C-enriched DNA (“heavy” fraction of treatments with and without isopod) to explore microbial interaction in response to *P. scaber*. Non-random co-occurrence analyses between fungal/bacterial OTUs were calculated using the Python module SparCC to determine correlations in the compositional data for network construction [60]. True SparCC correlations were selected based on a magnitude of >0.7 (positive correlations) or <−0.7 (negative correlations), with a statistical significance of *p* < 0.01. The *P*-values were obtained by 99 permutations of random selections of the data table. All networks were constructed with Gephi [61] in parallel, applying the same analytical pipeline for consistency. Network characteristics, also calculated in Gephi, were assessed based on their topology, that is, number of nodes (OTUs), edges (significantly correlated positive and negative connections), communities, modularity, average path length, network diameter, average degree, and clustering coefficient (Table 1). Additionally, the betweenness centrality, indicative of “key nodes” within the network, was identified. These nodes act as a bridge, connecting other nodes along the shortest path at a higher frequency [62, 63]. The interpretation of the network topology is given in Table 1.

**Table 1:** Topological properties of the co-occurrence network analysis derived from the ^13^C-enriched ITS and 16 S rRNA gene in the microcosms with and without *P. scaber*.

Network properties	+ isopod + leaf litter	− Isopod + leaf litter
Number of nodes^a^	444	317
Number of edges^b^	2028	1027
Positive edges^c^	1250 (62%)	639 (62%)
Negative edges^d^	778 (38%)	388 (38%)
Bacteria-Bacteria	1249 (62%)	329 (32%)
Bacteria-Fungi	678 (33%)	514 (50%)
Fungi-Fungi	101 (5%)	184 (18%)
Modularity^e^	2.26	2.30
Number of communities^f^	55	47
Network diameter^g^	13	7
Average path length^h^	5.04	2.17
Average degree^i^	9.13	3.24
Av. clustering coefficient^j^	0.385	0.144

^a^Microbial taxon (at genus level) with at least one significant (*P* < 0.01) and strong (SparCC > 0.7 or < − 0.7) correlation.

^b^Number of connections/correlations obtained by SparCC analysis.

^c^SparCC positive correlation (>0.7 with *P* < 0.01).

^d^SparCC negative correlation (< − 0.7 with *P* < 0.01).

^e^The capability of the nodes to form highly connected communities, that is, a structure with high density of between nodes connections (inferred by Gephi).

^f^A community is defined as a group of nodes densely connected internally (Gephi).

^g^The longest distance between nodes in the network, measured in number of edges (Gephi).

^h^Average network distance between all pair of nodes or the average length off all edges in the network (Gephi).

^i^The average number of connections per node in the network, that is, the node connectivity (Gephi).

^j^How nodes are embedded in their neighborhood and the degree to which they tend to cluster together (Gephi).

### Statistical analysis

The level of significance (*p* < 0.01) in the gene abundances (ITS, 16 S rRNA and *pmoA* gene), and methane uptake and carbon dioxide production rates between treatments, or over time was determined using ANOVA in Sigmaplot version 12.5 (Systat Software Inc., USA) after testing the data for normal distribution (Kolmogorov-Smirnov and Shapiro-Wilk test).

## Results

### Response of the methane and carbon dioxide fluxes to the presence of P. scaber

**Fig. 1:**
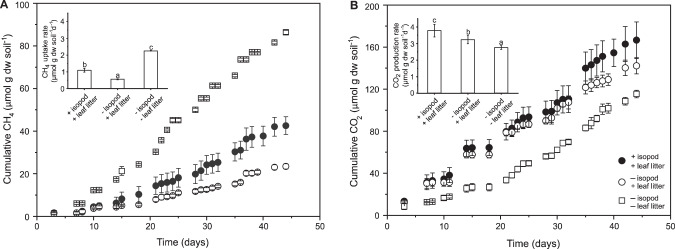
Cumulative methane uptake and carbon dioxide production. Cumulative methane uptake (**A**) and carbon dioxide production (**B**) in microcosms with and without isopods (mean ± s.d.; *n* = 6), and in the reference (mean ± s.d.; *n* = 4). Inset figures depict the culmulative methane uptake rate in (**A**), and cumulative carbon dioxide production rate in (**B**). The letters indicate the level of significance between treatments at *p* < 0.01.

Cumulative methane uptake was significantly (*p* < 0.01) higher in the presence of *P. scaber* and leaf litter, compared to microcosms without the isopods and leaf litter (Fig. 1). However, methane uptake was significantly (*p* < 0.01) lower in these microcosms than in the reference containing only soil, suggesting that the leaf litter adversely affected the methanotrophic activity (Fig. 1). Cumulative carbon dioxide production was significantly (*p* < 0.01) higher in the presence of *P. scaber* than in the other microcosms, whereas the least carbon dioxide accumulated in the reference (Fig. 1). The increase in cumulative methane uptake (and carbon dioxide production) detected in the microcosm containing *P. scaber* in γ-irradiated soil during incubation suggests that the isopod-derived methanotrophs were active and potentially able to re-colonize the soil (Fig. S2).

### Response of the fungal and bacterial marker gene abundances to the presence of P. scaber

**Fig. 2:**
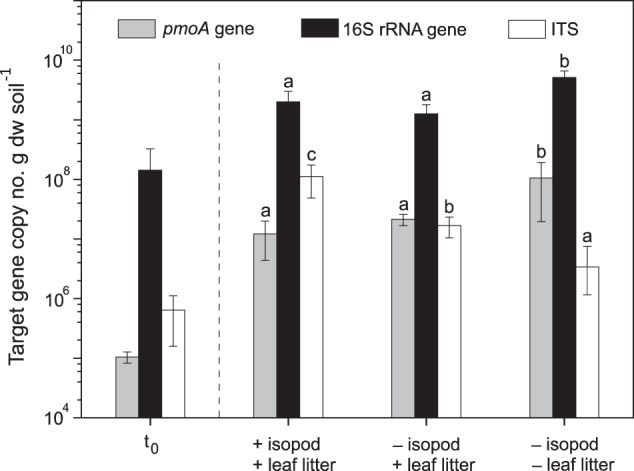
The abundance of the bacterial and fungal communities. The abundances of the *pmoA*, 16 S rRNA, and ITS genes at the start (t_0_), and end of the incubation (44 days) with and without *P. scaber*, and in the reference. Duplicate qPCR reactions were performed per replicate (*n* = 4 or 6), gene, and time. The letters indicate significant differences (*p* < 0.01) between treatments independently for the *pmoA*, 16 S rRNA, and ITS genes after the incubation.

The change in the fungal, total bacteria, and methanotroph abundances during the incubation were followed by enumerating the ITS, 16 S rRNA, and *pmoA* genes, respectively, in the starting material and after incubation. The ITS copy numbers appreciably increased by one to three orders of magnitude after the incubation (Fig. 2). Specifically, the ITS abundance was significantly (*p* < 0.01) higher in the presence of *P. scaber*, and lowest in the reference microcosm after the incubation (Fig. 2). In contrast, the 16 S rRNA gene abundance was significantly (*p* < 0.01) higher in the reference microcosm after the incubation than in the other microcosms, which showed comparable values (Fig. 2). Although the mean *pmoA* gene abundance was lower in the presence of *P. scaber* (1.2 × 10^7^ gene copy numbers g dw soil^−1^), gene copy numbers were within a narrow range comparable to the values in the microcosm without the isopod (2.1 × 10^7^ gene copy numbers g dw soil^−1^; Fig. 2). Generally, the abundances of the ITS, 16 S rRNA, and *pmoA* genes increased in the microcosms containing *P. scaber* in γ-irradiated soil, more pronounced for the fungal population which increased by approximately three orders of magnitude during the 49-day incubation (Fig. S3).

### Response of the metabolically active fungal and bacterial community composition to the presence of P. scaber, as determined by DNA-based SIP

A density gradient (~1.76 to ~1.66 g ml^−1^) was obtained after fractionation, showing a clear separation of the ^13^C- and ^12^C-DNA fractions (i.e., “heavy” and “light” fractions; Fig. S1). Subsequently, the ITS and 16 S rRNA gene from the “heavy” and “light” fractions of the ^13^C-CH_4_ incubations and the “light” fraction of the ^12^C-CH_4_ incubations were sequenced. Sequence analysis revealed a generally distinct community composition in the “heavy” and “light” fractions, supporting the density gradient fractionation (Fig. S4). Further ordination (RDA) and co-occurrence network analyses were performed considering the fungal and bacterial communities derived from the “heavy” fraction, representing the methane-dependent metabolically active and replicating community members.

The response of the active bacterial community composition to the presence of *P. scaber* is revealed in the PCA, showing a clear separation of the community in the incubations with and without isopods along PC axis 2 (explaining 15.15% of the total variance; Fig. S4). The active bacterial community was overwhelmingly represented by OTUs affiliated to Proteobacteria and Actinobacteria, together representing approximately 90% of the total community (Fig. S4). Similarly, the active fungal community was distinct in the presence of isopods, and could be separated along PC axis 1 (explaining 21.44% of the total variance in the PCA) from the community without the isopods (Fig. S4). The active fungal community was predominantly comprised of members affiliated to Ascomycota and Basidiomycota, representing approximately 90% of the total community. The bacteria and fungi identified as key nodes (i.e., nodes with high betweenness centrality) at finer taxonomic resolution (genus/species-level) were further explored (see below, Table 2).

**Table 2:**
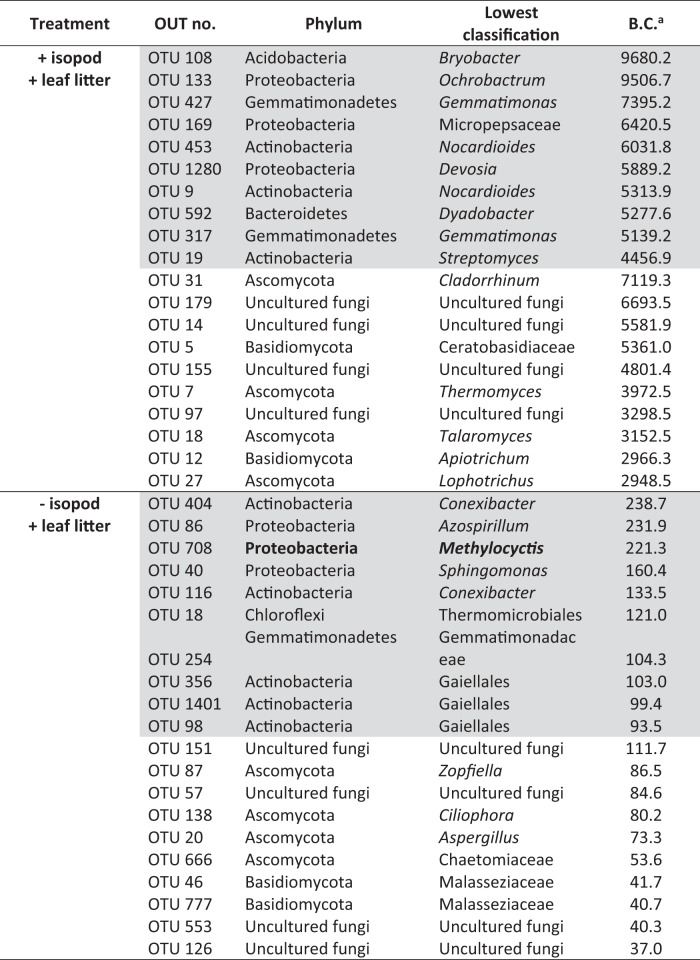
Top 10 bacterial (shaded gray) and fungal key nodes at the OTU level with more betweenness centrality (B.C.) in the microcosms with and without *P. scaber*.

Methanotroph-affiliated key node is emboldened.

**Fig. 3:**
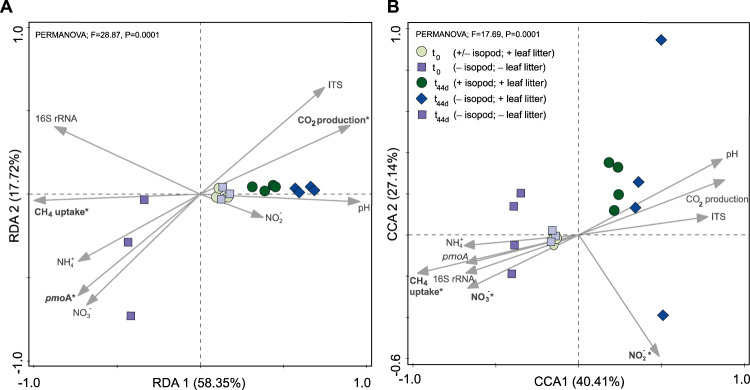
Response of the microbial community to the abiotic environment. RDA showing the response of the metabolically active (i.e., ^13^C-DNA-derived) bacterial (**A**) and fungal (**B**) community composition to the environmental parameters (cumulative methane uptake and carbon dioxide production rates, pH, ammonium, nitrate, nitrite, as well as the ITS, 16 S rRNA and *pmoA* gene abundances) in the different treatments (reference, with and without *P. scaber*). The asterisk indicates the level of significance (*p* < 0.01) of the environmental parameters.

Integrating the activity measurements (cumulative methane uptake and carbon dioxide fluxes) and bacterial/fungal compositional data after the incubation in an RDA, revealed the variables affecting the community composition. Among these variables, cumulative methane uptake and carbon dioxide production, as well as the *pmoA* gene abundance significantly (*p* = 0.0001) correlated to the bacterial community composition, whereas the composition of the fungal community were significantly (*p* = 0.0001) related to the cumulative methane uptake, nitrite, and nitrate (Fig. 3). Not surprisingly, methane uptake was positively correlated to both the bacterial and fungal communities in the reference, given that methane was the sole carbon source (besides indigenous soil carbon) in these incubations and that methane uptake was not inhibited in the reference. The RDA also revealed shifts in the bacterial and fungal community composition, comparing the reference (- isopods and - autoclaved leaf litter) to the incubations with and without isopods (Fig. 3). The microcosms supplemented with the autoclaved leaf litter (+ and – isopod) appear to harbor a more similar community composition than in the reference without the leaf litter; in the absence of the reference in the community analysis, the incubations containing autoclaved leaf litter with and without *P. scaber* could be clearly separated (Figure S4). Thus, *P. scaber* induced a shift in the bacterial and fungal community composition after the incubation (44 days) in the presence of the leaf litter.

### Response of the methane-driven fungal and bacterial interaction network to the presence of P. scaber, as determined by SIP-network analysis

**Fig. 4:**
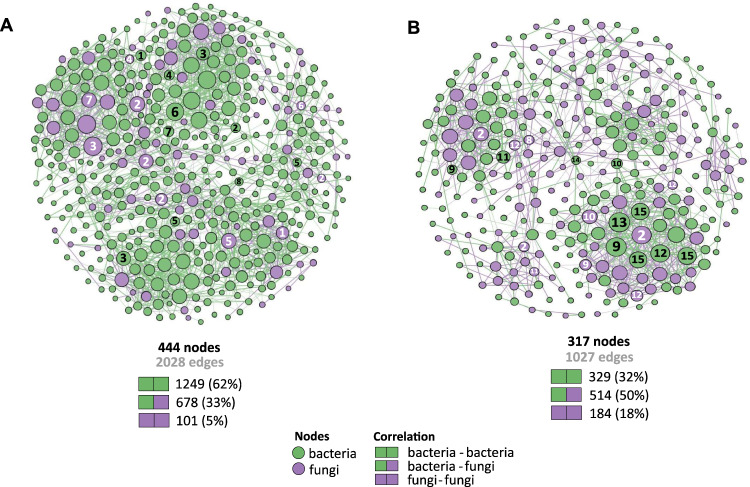
Co-occurrence network analysis of the metabolically active microbial community. Co-occurrence network analysis derived from the metabolically active bacterial and fungal communities in the microcosms with (**A**) and without (**B**) *P. scaber*. The topological properties of the networks are given in Table 1. Each node indicates a bacterial (green) or fungal (purple) taxon at the OTU level. The SparCC correlations were based on a magnitude of >0.7 (positive correlation) or <− 0.7 (negative correlation), and is statistically significant (*P* < 0.01). The size of the nodes is proportional to the number of connections (degree), and the top key nodes (i.e., nodes with the highest betweenness centrality) for bacteria and fungus are numbered, with the betweenness centrality values given in Table 2. The bacterial key nodes, given to the finest taxonomic resolution where available, are: (1) *Bryobacter*; (2) *Ochrobactrum*; (3) *Gemmatimonas*; (4) Micropepsaeae; (5) *Nocardioides*; (6) *Devosia*; (7) *Dyadobacter*; (8) *Streptomyces*; (9) *Conexibacter*; (10) *Azospirillum*; (11) ***Methylocystis***; (12) *Sphingomonas*; (13) Thermomicrobiales; (14) Gemmatimonadaceae; (15) Gaiellales. The fungal key nodes, given to the finest taxonomic resolution where available, are: (1) *Cladorrhinum*; (2) Uncultured fungi; (3) Ceratobasidiaceae; (4) *Thermomyces*; (5) *Talaromyces*; (6) *Apiotrichum*; (7) *Lophotrichus*; (8) *Zopfiella*; (9) *Ciliophora*; (10) *Aspergillus*; (11) Chaetomiaceae; (12) Malasseziaceae. Nodes representing methanotrophs are emboldened.

A co-occurrence network analysis derived from the ^13^C-enriched DNA was performed to determine the response of the microorganisms involved in the trophic interaction, comprising of the bacterial and fungal communities, to *P. scaber* bioturbation. The microbial network was assessed based on the topological properties, comparing the microcosms with and without *P. scaber* (Table 1). The network analysis revealed a more connected and complex community in the presence than absence of isopods, as indicated by the higher number of nodes (microbial taxa, OTU level), edges (number of connections or correlations), degree (number of edges per node), and clustering coefficient (tendency to cluster together) (Table 1). Focusing on the number of connections, the presence of isopods resulted in a higher proportion of bacteria-bacteria interaction, whereas the share of fungal interactions (i.e., both bacteria-fungus and fungus-fungus interactions) decreased when compared to the network without isopods, albeit fungal abundance significantly increased after the incubation (Fig. 4 and Table 1). Accordingly, modularity (ability to compartmentalize or form densely connected nodes within the network) was comparable in both treatments. With a more complex network in the presence of isopods, we documented closer association among active members of the methane-driven community, in response to *P. scaber* activity.

The top 10 key nodes (i.e., nodes with the highest betweenness centrality) belonging to the bacterial and fungal communities were identified (Table 2 and Fig. 4). *Methylocystis*-affiliated (alphaproteobacterial methanotroph) OTU was detected as a bacterial key node in the microcosm without *P. scaber*, whereas the top 10 key nodes in the microcosm containing the isopods exclusively comprised of non-methanotrophs, indicating that the non-methanotrophs may become relevant during perturbation. The bacterial key nodes were distinct in both treatments (+ and – isopods), and were represented by non-methanotrophs typically detected alongside the methanotrophs in methane-enriched environments (e.g., *Sphingomonas*, *Conexibacter*, *Gemmatimonas*, Gemmatimonadaceae; [28, 38]). The fungus-affiliated key nodes were represented by Ascomycota and Basidiomycota, with distinct community members forming the key nodes in the microcosms with and without the isopod (Table 2). Many of these fungal species are saprotrophs, and are fast-growers (e.g., yeasts). It thus appears that methane-derived carbon fueled key bacterial and fungal members of the community.

## Discussion

### P. scaber-induced effects on the methanotrophic activity, microbial respiration, and the abiotic environment

*P. scaber* bioturbation significantly stimulated the methane uptake rates and/or relieved the inhibitory effects of the leaf litter. However, the positive effect was not reflected in the methanotroph abundance, where the *pmoA* gene copy numbers were in the same order of magnitude in the microcosms with and without the isopod (Fig. 2). More likely, *P. scaber* induced the apparent cell-specific methanotrophic activity, instead of stimulating methanotroph growth and proliferation, which can be supported by quantifying the *pmoA* gene transcripts. Alternatively, having comparable *pmoA* gene copy numbers in + and – isopod treatments, despite significantly higher methane uptake in the presence of isopods may be attributable to a high turnover of the methanotroph biomass, and/or that the activity of the sMMO (soluble methane monooxygenase) in methanotrophs containing both pMMO and sMMO may have been overlooked, albeit methanotrophs containing only the sMMO could not be detected in this soil [39, 41]. Intriguingly, the presence of the autoclaved leaf litter adversely affected methane uptake, but the inhibitory effect was more severe in the absence of *P. scaber* (Fig. 1). Indeed, methane uptake can be inhibited by plant-derived monoterpenes, as shown in a forest soil [64]. Although the volatile compounds emitted from the leaf litter were below the detection limit (data not shown), we detected comparably higher cymol (belonging to the monocyclic monoterpenes, like terpinene and limonene known to inhibit methane oxidation; [64, 65]), dill ether, and 2-phenyl-2-propanol in the incubations containing the leaf litter, when compared to atmospheric concentrations of these compounds. This indicates that the autoclaved leaf litter still emits trace levels of volatile compounds, sufficient to inhibit the methanotrophs. Nevertheless, the inhibitory effects of these compounds could be alleviated by the actions of the isopods (comminution, physical re-organization of the soil), which may in part, increase soil aeration [3], in turn, promotes aerobic processes, including methane oxidation. Improved soil aeration could be confirmed using oxygen microsensors (e.g., [46]) in future work. In addition to heightened soil methane uptake, previous work documented the stimulatory effect of macroinvertebrates on specific fungal and bacterial activities (e.g., oxygenases, phenol oxidases, glucosidases activity; [66–69]). Taken together, we found evidence supporting our hypothesis showing increased methanotrophic activity in the presence of *P. scaber*, but refuting our hypothesis, the increase was not sustained by higher methanotrophic growth.

As anticipated, the addition of autoclaved leaf litter significantly stimulated microbial respiration, as indicated by the higher carbon dioxide production in treatments with leaf litter when compared to those in the reference microcosm without the extraneous carbon source. The supplemented autoclaved leaf litter did not affect the initial bacterial and fungal community composition, but shifted the trajectory of community succession over time (44 days), comparing the reference to the incubations without *P. scaber* (Fig. 3). The leaf litter thus strongly affected the activity and composition of the soil-borne microorganisms. Nevertheless, *P. scaber*-induced effects were still discernable.

Among the abiotic parameters, ammonium concentration was significantly higher in the presence of *P. scaber*. Isopod activity may increase ammonium mobilization or mineralization, resulting in the higher ammonium concentrations, as well as other inorganic nitrogen (nitrite and nitrate), coinciding with the elevated carbon dioxide production (Figs. 1, S5). Alternatively, *P. scaber* also releases ammonium via fecal deposits [4]; this was more pronounced in the microcosms containing the isopods in γ-irradiated soil where ammonium concentrations increased over time (Fig. S5). It follows that the products of ammonium oxidation (e.g., nitrite and nitrate) may also affect the microbial community, as indicated in the RDA (Fig. 3). Regardless of the source, increased ammonium availability may relieve nitrogen limitation, and stimulated the methanotrophic activity under excess methane availability (Fig. 1; [48, 70, 71]), but this postulation needs further investigation. Previously, elevated methane uptake could be correlated to improved nitrogen supply in earthworm-impacted soils [16]. Besides nitrogen, the activity of the isopods, along with other macroinvertebrates, have been shown to liberate organic carbon, as well as cations (magnesium and calcium) in litter-amended soil [7]. Summarized, *P. scaber* engineers its habitat, significantly influencing the methanotrophic activity, microbial respiration, and the soil nutritional status.

### P. scaber-induced effects on the metabolically active bacteria and fungi

*P. scaber* differentially affected the microbial population, significantly stimulating the fungal abundance concomitant to a shift in the community composition, while having marginal effects on the bacterial abundances, as revealed in the qPCR and community analyses. *P. scaber*-induced stimulation of the fungal abundance is in line with previous work, showing significant increase in the fungal biomass when macroinvertebrates were present [66]. Compared to microinvertebrates (e.g., nematodes, protozoa), it appears that *P. scaber* does not selectively graze on fungi [72, 73]. Although the bacterial abundance was not significantly affected, a compositional shift was documented after the incubation in the presence of *P. scaber* (Figs. 3 and S4). By releasing and/or re-distributing resources (increase labile C and N availability; [66]), as a result of bioturbation, the isopod may thus shape the composition of both the active bacterial and fungal communities over time.

### P. scaber-induced emergence of a more complex methane-driven bacteria and fungus interaction, as revealed by coupling SIP to a co-occurrence network analysis

A more complex and connected network emerged in the presence of *P. scaber*, demonstrating that the isopod activity fostered closer association of the interacting microbial community. Through comminution, soil aggregates are disrupted, and the soil as a habitat for the microorganisms are homogenized [74]. Soil mixing can thus increase the likelihood for interaction to occur, bridging microorganisms which otherwise would be physically apart, particularly for the immobile bacteria, as indicated by the higher bacteria-bacteria interaction (edges) in the *P. scaber*-impacted network (Table 1). With the significant stimulation of fungal abundance in the presence of *P. scaber* (Fig. 2), the fungal hyphae presumably facilitated the translocation of nutrients, as well as microorganisms [75–77], thereby re-distributing nutrients and bacteria across space. This likely promoted metabolic exchange, in turn increasing interaction complexity. Therefore, *P. scaber*-induced higher methanotrophic activity can be directly linked to the more complex network topology, with consequences for community function in soils colonized by the isopods.

Noteworthy, fungal ITS was retrieved from the ^13^C-enriched DNA, indicating that the methane-derived ^13^C was assimilated into fungi via trophic interaction with methanotrophs. Rarely has the interaction between methanotrophs and fungi been documented. Previously, *Methylocapsa acidiphilia* (an acidophilic alphaproteobacterial methanotroph capable of nitrogen fixation and methanol assimilation; [78]) was detected at high relative abundance alongside the white rot fungus *Hypholoma fasciculare* in a beech wood, while other bacteria were inhibited by the fungus-induced bactericidal effects [79]. It thus seems that this methanotroph was selectively enriched in the presence of *H. fascilulare*, prompting Ho and colleagues [37] to postulate that the methanotroph and fungus may mutually benefit from the interaction, where the methanotrophs act as a source of nitrogen, in return for carbon (methanol, a by-product of fungal ligninolytic activity). Besides the potential interaction between *M. acidiphilia* and *H. fascilulare*, other (trophic) interactions between methanotrophs and fungi are not known. Here, it remains to be determined whether assimilation of ^13^C into the fungal community occurred via passive (e.g., ^13^C obtained via dead and lysed methanotroph cells) or active (e.g., uni- and bi-directional flow of ^13^C substrate) transfer of substrate, deserving future attention.

Many key nodes were represented by the non-methanotrophs, including members of the fungal community, indicating their relevance in the methane-driven network, and possibly, playing a role in modulating methanotrophic activity [24, 28, 80, 81]. These key nodes may not necessarily be the most abundantly represented taxa, but connects other nodes at higher frequencies [63]; hence, systematic removal of the key nodes will unravel the network [82]. *Methylocystis* (alphaproteobacterial methanotroph) was identified as a key node in the microcosm without the isopod, corroborating previous work showing the predominance of these methanotrophs in the same soil [42]. Some identified non-methanotroph key nodes (e.g., *Gemmatimonas*, *Sphingomonas*) had been consistently detected in methane-driven networks from other environments (rice paddy soil, peatlands, river sediment; [24, 28, 38]). All fungal key nodes are known species of saprotrophs, and many were described as fast-growing fungi, including yeasts (*Apiotrichum, Talaromyces*, Malasseziaceae). In the case of *Thermomyces*, it has recently been shown that this fungus thrives in a co-culture with actinomycetes (bacteria) [83], which could explain their co-occurrence. Furthermore, many identified fungal key nodes in the presence of *P. scaber* are heat-tolerant, and often detected on surfaces of animals or in dung [84, 85]. There were, however, no known fungal pathogens or animal symbionts. Hence, the selection of the fungal key nodes could be associated to their life style (fast-growers) and/or heat tolerance, but the mechanism driving their co-occurrence with other bacteria in this study remains to be determined. Nevertheless, the fast-growing fungi may benefit from the supplemented leaf litter, decomposing the extraneous litter-derived lignocellulose together with the bacteria [86], which was accelerated by the activity of the isopod (i.e., via nutrient and oxygen supply). Taken together, both bacterial and fungal key nodes in the isopod-impacted community were distinct from those derived from the microcosm without the isopod, indicating that *P. scaber* affected the key community members.

### Microbial hitchhikers: P. scaber facilitation of microbial dispersal

*P. scaber* appears to be a dispersal agent for soil microorganisms. The increase in the fungal and bacterial abundances in the microcosms containing the isopod in γ-irradiated soil during the incubation was concomitant to carbon dioxide production. Although respiration by *P. scaber* may have masked microbial respiration, more conclusive evidence is provided by the methane flux measurements. The onset of methane uptake was detected after 3 days, indicating the presence of an active methanotrophic population, re-colonizing the γ-irradiated soil. Increased nutrient availability as a result of mineralization, accompanied by empty niches (space) following γ-irradiation may have spurred the growth of the methanotrophs under methane availability [41, 46, 87]. Likewise, the fungal community probably benefited from these resources, as indicated by the increase in the ITS abundance by three orders of magnitude in the presence of *P. scaber* (Figure S3). The isopods were lightly rinsed with autoclaved deionized water to dislodge loosely attached soil particles prior to microcosm set up, which cannot completely exclude the microorganisms that strongly adhere to the surface of *P. scaber*. Nevertheless, these microorganisms may represent epibionts. It remains to be determined whether the hitchhiking microorganisms that re-colonized the γ-irradiated soil were derived from the gut (through fecal deposition) [20]; and/or body surface of *P. scaber*, and whether the microorganisms were passively (i.e., unintentional dispersal) and/or actively (i.e., reciprocal selection of microorganism-macroinvertebrate) transported. Regardless of the source and exact transfer mechanism, it is evident that *P. scaber* facilitated microbial dispersal.

## Conclusion

We showed the fundamental relationship between macroinvertebrates, exemplified by *P. scaber* and the microbial community in an agricultural soil, and how the interkingdom interaction influenced ecosystem function. Confirming our hypothesis, *P. scaber* positively affected soil methane uptake, and fostered closer association among members of the methane-driven community. Admittedly, single-species macroinvertebrate effects on the bacterial and fungal communities were investigated in the present study. This is not without relevance, given that the identity or composition of the macroinvertebrate, rather than the diversity (richness), has been shown to be relatively more important in determining the effects of macroinvertebrates on soil ecosystem function [7]. Considering that soil isopods are typically present at high densities in their habitat, our study may also be applicable under field conditions, provided *P. scaber* predominate the same niche. Our findings reinforced previous work, showing the effects of the isopod on the edaphic properties, as well as the soil microbial communities and respiration; widening current understanding, we showed that *P. scaber* modulates the methanotrophic activity, and the interaction of the methane-oxidizing community, comprising of bacteria and fungus. More generally, research findings implied that the methane sink in agricultural soils can be strengthened by applying agricultural management practices that promote the soil isopods.

## Supplementary information

Supplementary Information

## Data Availability

All data generated or analysed during this study are included in this published article or in the Supplementary Information files.
