# Supplementary material for: Interkingdom interaction: the soil isopod Porcellio scaber stimulates the methane-driven bacterial and fungal interaction
Source: ISME Commun. 2023 Jun 24;3:62. doi: 10.1038/s43705-023-00271-3 (PMC10290665; doi:10.1038/s43705-023-00271-3)
Supplement: Supplementary file 1 — Supplementary Information [file 43705_2023_271_MOESM1_ESM.pdf]

Supplementary Information

Interkingdom interaction: the soil isopod *Porcellio scaber* stimulates the methane-driven bacterial and fungal interactions.

Tanja Heffner[1#], Semi A. Brami[1#], Lucas W. Mendes[2], Thomas Kaupper[1], S. Emilia Hannula[3], Anja Poehlein[4], Marcus A. Horn[1*], Adrian Ho[1*].

[1]Institute for Microbiology, Leibniz Universität Hannover, Herrenhäuser Str. 2, 30419 Hannover, Germany.

[2]Center for Nuclear Energy in Agriculture, University of São Paulo CENA-USP, Brazil.

[3]Department of Environmental Biology, Institute of Environmental Sciences, Leiden University, Einsteinweg 2, 2333CC Leiden, the Netherlands.

[4]Department of Genomic and Applied Microbiology and Göttingen Genomics Laboratory, Institute of Microbiology and Genetics, Georg-August University Göttingen, Grisebachstr. 8, D-37077 Göttingen, Germany.

Included:

- Supplementary tables.
- Supplementary figure legends.

Supplementary tables

Table S1: Number of replaced dead isopods in each replicate (44-day incubation; + leaf litter, + isopod).

	Replicate	No. of isopods replaced
$^{12}C\text{-}CH_4$	1	10
	2	9
$^{13}C\text{-}CH_4$	1	12
	2	12
	3	8
	4	7

Table S2: Number of replaced dead isopods in each replicate (14- and 49-day incubation; + leaf litter, + isopod in γ-irradiated soil).

	Replicate	No. of replacements
Sampling after 14 days	1	2
	2	0
	3	2
Samling after 49 days	1	3
	2	4
	3	5

Supplementary figure legends

Figure S1: Relative *pmoA* gene abundance along the density gradient after fractionation in the ^{13}C- (mean ± s.d.; n=4) and ^{12}C-methane (mean ± s.d.; n=2) incubations with (A) and without (B) *P. scaber*. The relative abundance of the *pmoA* gene was determined from the proportion of each fraction over the sum of each sample. The arrows indicate the "light" (white) and "heavy" (black) fractions. ITS and 16S rRNA gene sequencing were performed for the "light" and "heavy" fractions of the ^{13}C-methane incubation, and the "light" fraction of the ^{12}C-methane incubation (see Figure S4).

Figure S2: Cumulative methane uptake (A) and carbon dioxide production (B) in microcosms containing *P. scaber* in γ-irradiated soil (mean ± s.d.; n= 3).

Figure S3: Temporal changes in the *pmoA*, 16S rRNA, and ITS gene abundances in the microcosm containing *P. scaber* in γ-irradiated soil (mean ± s.d.; n= 6). Duplicate qPCR reactions were performed per replicate (n=3), gene, and time. The s.d. for the *pmoA* gene is within the symbols.

Figure S4: The bacterial (A,C) and fungal (B,D) community composition in the "light" and "heavy" fractions of the ^{13}C-methane incubation (n=4), and the "light" fraction of the ^{12}C-methane incubation (n=2) with and without *P. scaber*. Compositional differences in community (relative abundance; C,D) is visualized in the PCA (A, B).

Figure S5: Temporal changes in the soluble ammonium concentration in the incubations with and without *P. scaber*, and reference in the starting material (t_0 sample) and after incubation (A), as well as in the incubation containing *P. scaber* in γ-irradiated soil (B). Other inorganic N (nitrate and nitrite) concentrations were below the detection limit.

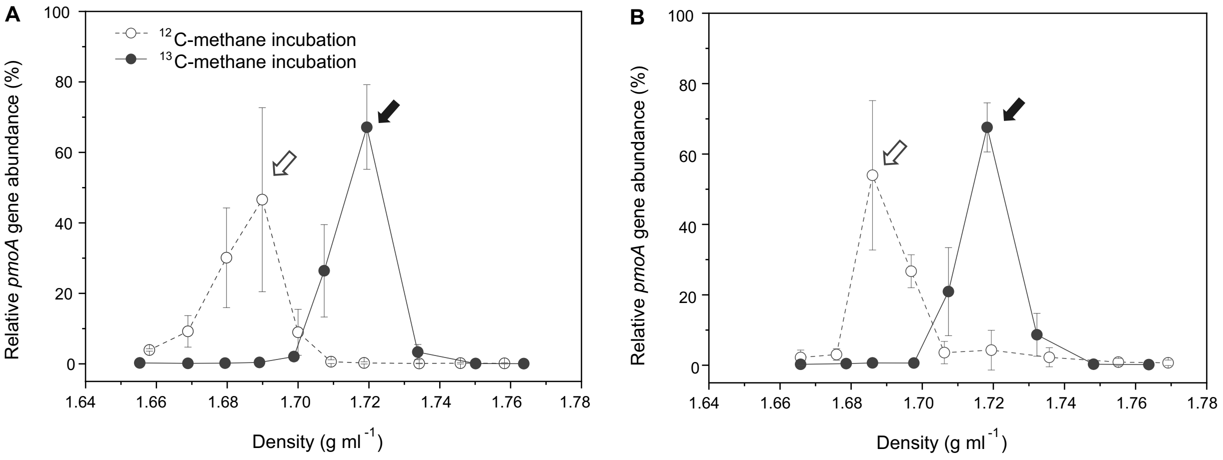
A
B
12C-methane incubation
13C-methane incubation
Relative pmoA gene abundance (%)
Density (g ml -1)
0
20
40
60
80
100
1.64
1.66
1.68
1.70
1.72
1.74
1.76
1.78

Figure S1

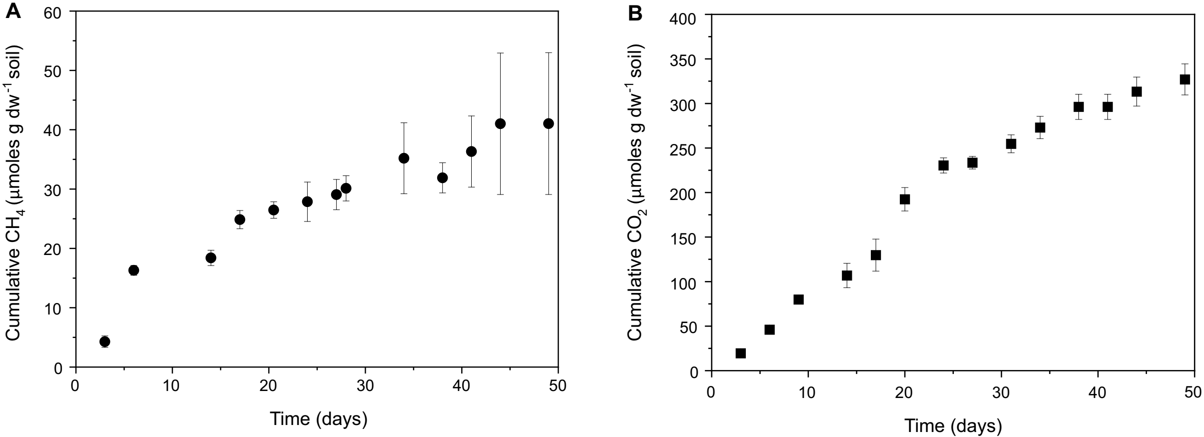

A
Cumulative CH4 (µmoles g dw-1 soil)
Time (days)
B
Cumulative CO2 (µmoles g dw-1 soil)
Time (days)

Figure S2

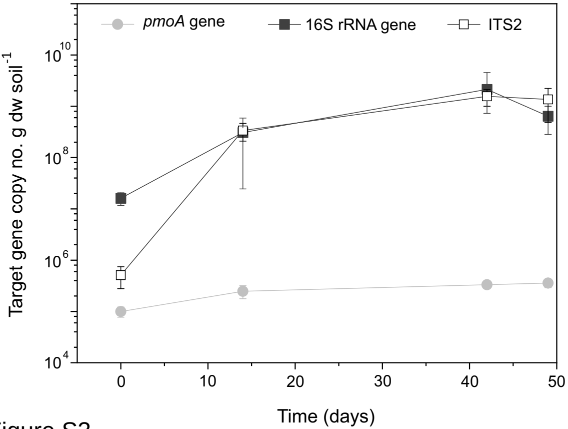
pmoA gene
16S rRNA gene
ITS2
Target gene copy no. g dw soil⁻¹
10^10
10^8
10^6
10^4
0
10
20
30
40
50
Time (days)

Figure S3

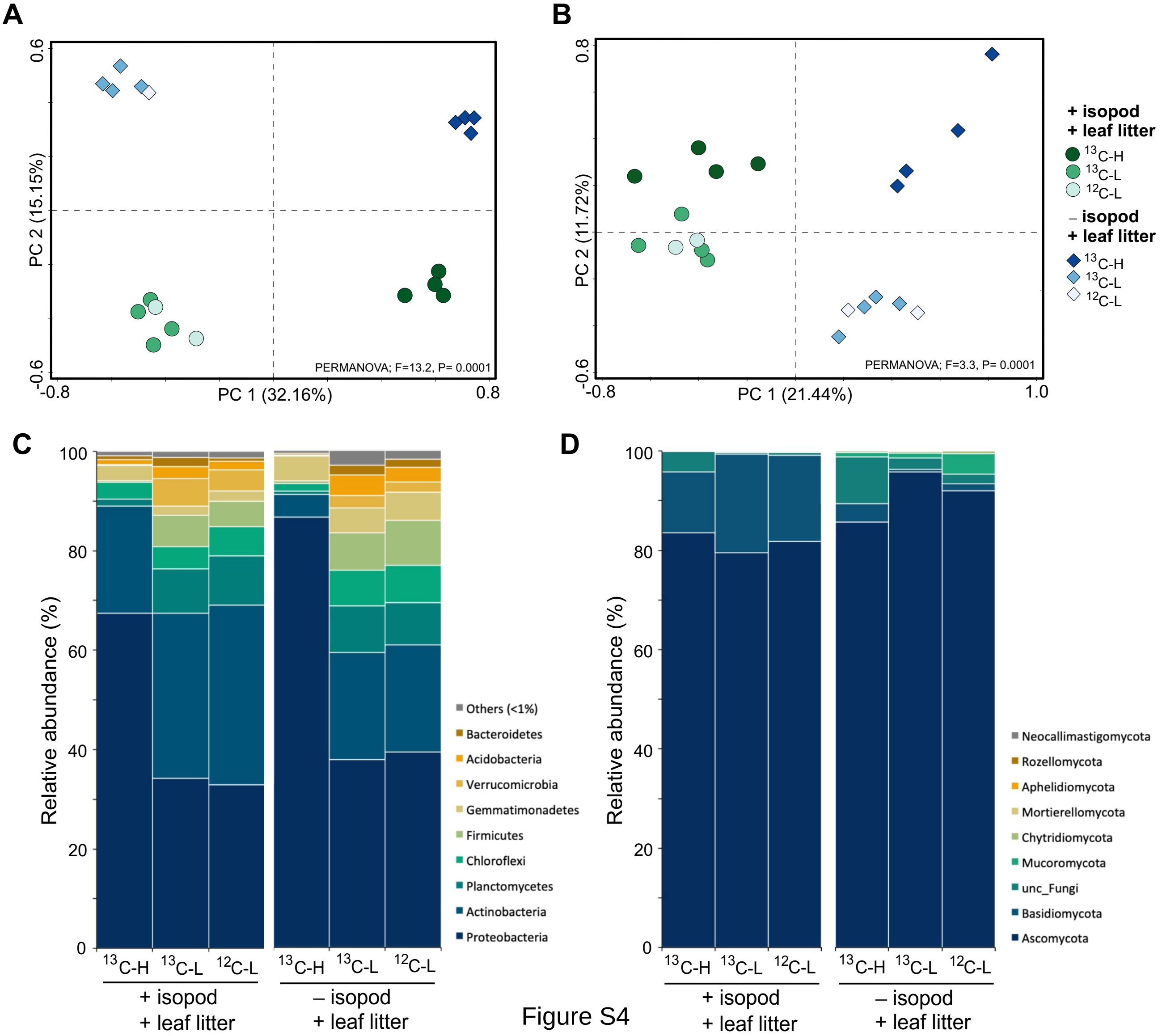
A
B
PC 2 (15.15%)
PC 1 (32.16%)
PERMANOVA; F=13.2, P= 0.0001
PC 2 (11.72%)
PC 1 (21.44%)
PERMANOVA; F=3.3, P= 0.0001
+ isopod
+ leaf litter
13C-H
13C-L
12C-L
– isopod
+ leaf litter
C
D
Relative abundance (%)
Others (<1%)
Bacteroidetes
Acidobacteria
Verrucomicrobia
Gemmatimonadetes
Firmicutes
Chloroflexi
Planctomycetes
Actinobacteria
Proteobacteria
Neocallimastigomycota
Rozellomycota
Aphelidiomycota
Mortierellomycota
Chytridiomycota
Mucoromycota
unc_Fungi
Basidiomycota
Ascomycota

Figure S4

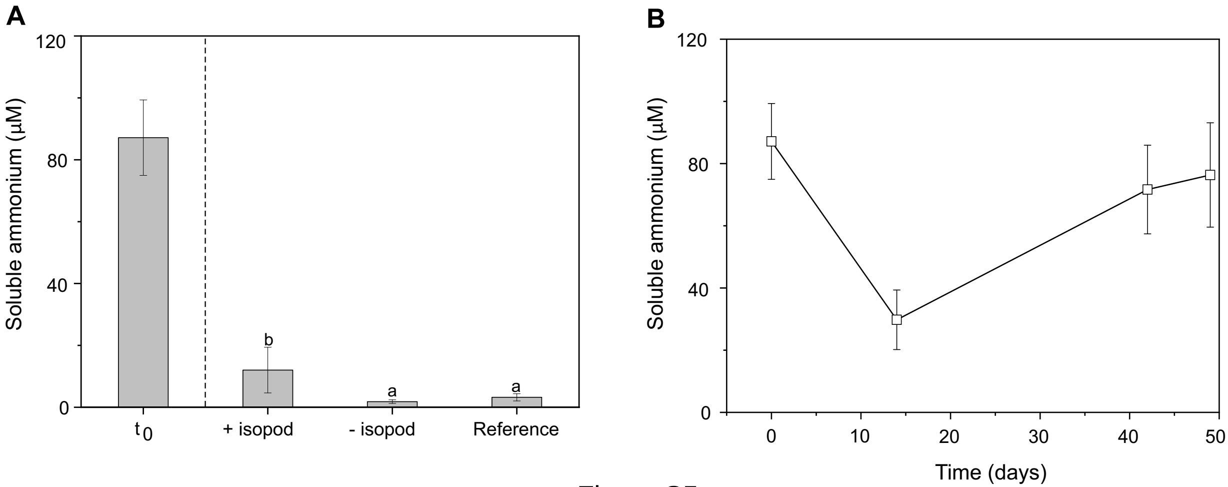
A
B
Soluble ammonium (µM)
120
80
40
0
t 0
+ isopod
- isopod
Reference
b
a
a
Soluble ammonium (µM)
120
80
40
0
0
10
20
30
40
50
Time (days)

Figure S5
